# 2269. Inpatient Antibiotic Prescribing for Symptomatic COVID-19 Patients in a Large US Sample, 2020-2022

**DOI:** 10.1093/ofid/ofad500.1891

**Published:** 2023-11-27

**Authors:** Michael Pulia, Meggie Griffin, Daniel J Hekman, Rebecca Schwei, Aurora E Pop-Vicas, Lucas Schulz, Meng-Shiou Shieh, Penelope Pekow, Peter Lindenauer

**Affiliations:** University of Wisconsin-Madison, Madison, Wisconsin; University of Wisconsin - Madison, Madison, Wisconsin; University of Wisconsin - Madison, Madison, Wisconsin; University of Wisconsin-Madison, Madison, Wisconsin; University of Wisconsin School of Medcine and Public Health, Madison, WI; University of Wisconsin Hospital and Clinics, Madison, Wisconsin; University of Massachusetts Chan Medical School-Baystate, Springfield, Massachusetts; University of Massachusetts Chan Medical School-Baystate, Springfield, Massachusetts; University of Massachusetts Chan Medical School-Baystate, Springfield, Massachusetts

## Abstract

**Background:**

Significant concerns have been raised regarding antibiotic exposure in patients with COVID-19 and the impact of the pandemic on antimicrobial stewardship in the acute care setting. Therefore, we assessed trends in inpatient antibiotic prescribing practices for symptomatic COVID-19 patients and patients with non-COVID-19 respiratory tract infections (RTIs).

**Methods:**

We identified non-elective, inpatient and observation admissions from March 2020 – December 2022 for COVID-19 and non-COVID-19 RTIs at 765 hospitals in the Premier Healthcare Database using an ICD-10 code algorithm. We described antibiotic usage as the % of encounters with at least one antibiotic dose and by highest level of care (general care, intermediate care (IMC), intensive care unit (ICU)) and antibiotic appropriateness (inappropriate, appropriate) which was characterized as the cooccurrence of ICD-10 code(s) indicating a bacterial infection, as defined by the CDC. We used chi-squared tests to compare antibiotic usage by level of care and by year for 2020 v. 2021 and 2021 v. 2022.

**Results:**

Our sample included 1,198,687 symptomatic COVID-19 cases and 1,713,714 non-COVID-19 RTIs. Antibiotic prescribing for COVID-19 declined from 75.6% in 2020 to 65.2% in 2021 (p < 0.001) and remained at 65.0% in 2022 (p = 0.093) (Fig. 1a). Non-COVID-19 RTIs had a significant decrease in antibiotic prescribing from 90.9% in 2020 to 88.1% in 2021 (p < 0.001) and 85.4% in 2022 (p < 0.001) (Fig. 1a). Inappropriate antibiotic prescribing for COVID-19 declined from 31.2% in 2020 to 27.4% in 2021 (p < 0.001) and 18.1% in 2022 (p < 0.001) (Fig. 1b). Antibiotic usage was significantly different for ICU, IMC, and general care for both COVID-19 (p < 0.001) (Fig. 1c) and non-COVID-19 RTIs (p < 0.001) (Fig. 1d).

**Figure 1.**
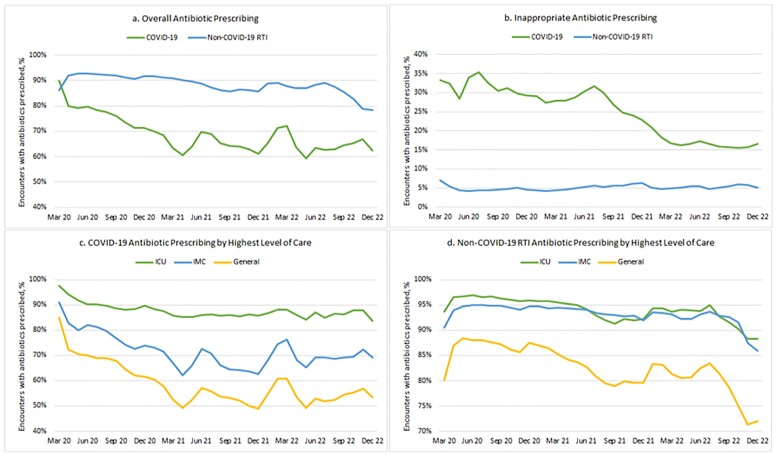
Monthly antibiotic prescribing trends for COVID-19 and non-COVID-19 RTI hospital encounters

**Conclusion:**

Despite downward trends in antibiotic prescribing, a majority of COVID-19 patients are prescribed an antibiotic at some point during their admission. Our analysis suggests most of these patients have a co-occurring appropriate indication for antibiotics, yet nearly 1 in 5 COVID-19 patients still receive non-indicated antibiotics. Future work should identify drivers of inappropriate antibiotic prescribing in COVID-19 patients.

**Disclosures:**

**All Authors**: No reported disclosures

